# GLORI: A GNSS-R Dual Polarization Airborne Instrument for Land Surface Monitoring

**DOI:** 10.3390/s16050732

**Published:** 2016-05-20

**Authors:** Erwan Motte, Mehrez Zribi, Pascal Fanise, Alejandro Egido, José Darrozes, Amen Al-Yaari, Nicolas Baghdadi, Frédéric Baup, Sylvia Dayau, Remy Fieuzal, Pierre-Louis Frison, Dominique Guyon, Jean-Pierre Wigneron

**Affiliations:** 1 CESBIO, Université de Toulouse, CNES/CNRS/IRD/UPS, Toulouse, France18 Avenue Edouard Belin, Toulouse Cedex 9 31401, France; mehrez.zribi@cesbio.cnes.fr (M.Z.); pascal.fanise@cesbio.cnes.fr (P.F.); frederic.baup@cesbio.cnes.fr (F.B.); fieuzalr@cesbio.cnes.fr (R.F.); 2 Cooperative Institute for Climate and Satellites, University of Maryland (CICS-MD/UMD), Appointed at the Laboratory for Satellite Altimetry, National Oceanographic and Atmospheric Administration (NOAA), College Park, MD 20740, USA; alejandro.egido@noaa.gov; 3 GET, Université de Toulouse, CNRS, IRD, UPS, 14 Avenue Édouard Belin, Toulouse 31400, France; jose.darrozes@get.obs-mip.fr; 4 ISPA, INRA, Bordeaux Sciences Agro, Villenave d’Ornon 33140, France; amen.alyaari@bordeaux.inra.fr (A.A.-Y.); sylvia.dayau@bordeaux.inra.fr (S.D.); guyon@bordeaux.inra.fr (D.G.); wigneron@bordeaux.inra.fr (J.-P.W.); 5 IRSTEA, UMR TETIS, 500 Rue François Breton, Montpellier cedex 5 34093, France; nicolas.baghdadi@teledetection.fr; 6 Matis-IGN/UPEM, 5 Boulevard Descartes, Marne la Vallée Cedex 2 77454, France; pierre-louis.frison@u-pem.fr

**Keywords:** soil moisture, vegetation, biomass, airborne, microwave, L-Band, GPS, reflectometry, multistatic radar, GNSS-R

## Abstract

Global Navigation Satellite System-Reflectometry (GNSS-R) has emerged as a remote sensing tool, which is complementary to traditional monostatic radars, for the retrieval of geophysical parameters related to surface properties. In the present paper, we describe a new polarimetric GNSS-R system, referred to as the GLObal navigation satellite system Reflectometry Instrument (GLORI), dedicated to the study of land surfaces (soil moisture, vegetation water content, forest biomass) and inland water bodies. This system was installed as a permanent payload on a French ATR42 research aircraft, from which simultaneous measurements can be carried out using other instruments, when required. Following initial laboratory qualifications, two airborne campaigns involving nine flights were performed in 2014 and 2015 in the Southwest of France, over various types of land cover, including agricultural fields and forests. Some of these flights were made concurrently with *in situ* ground truth campaigns. Various preliminary applications for the characterisation of agricultural and forest areas are presented. Initial analysis of the data shows that the performance of the GLORI instrument is well within specifications, with a cross-polarization isolation better than −15 dB at all elevations above 45°, a relative polarimetric calibration accuracy better than 0.5 dB, and an apparent reflectivity sensitivity better than −30 dB, thus demonstrating its strong potential for the retrieval of land surface characteristics.

## 1. Introduction

Physical soil and vegetation characteristics play an essential role in the functioning of continental water and carbon cycles [1,2]. Over the last three decades, active microwave remote sensing techniques have been used to retrieve soil moisture and vegetation biomass [3,4,5]. In addition, various theoretical and experimental inversion techniques have been proposed for the retrieval of land surface parameters, allowing for the monitoring of their spatial and temporal variations. Despite the implementation of many technical and algorithmic improvements, and the development of numerous radar and radiometer constellations, operational applications remain small in number, in particular as a consequence of coverage and/or resolution limitations.

Global Navigation Satellite System reflectometry (GNSS-R) is a remote sensing technique based on the analysis of L-band GNSS signals transmitted by navigation constellations such as the Global Positioning System (GPS) following their reflection from the Earth’s surface [6]. GNSS-R remote sensing has several advantages. The first of these arises from the full-time, global coverage provided by GNSS satellites—their continuously transmitted signals provide an opportunity for the relatively straightforward implementation of very dense, multi-static L-band radar measurements. Secondly, the operating principle of GNSS-R does not require the use of radar transmitters, other than those already provided by the GNSS satellites. This allows for the development of light, compact and inexpensive receiving systems. Thirdly, the nature of circularly polarized L-band signals allows this technique to be used under all weather conditions, and makes it suitable for the sensing of a wide range of surface parameters.

Over the last 20 years, experimental and theoretical studies made with GNSS-R have demonstrated its true potential for the evaluation of Earth surface characteristics. Although this technique was first developed for the measurement of sea surface parameters such as height [6] and roughness [7], other more recent studies have focused on the monitoring of land surface characteristics such as soil moisture [8,9], fraction of vegetation cover [10,11], forest biomass [12,13], the mapping of flooded areas [14], and the detection of snow or ice [15,16]. Experimental campaigns from both fixed [17,18,19], airborne [13,20,21,22], balloon [23] and more recently from Low Earth Orbit [24] platforms have confirmed the sensitivity of GNSS receivers to soil and biomass characteristics. In parallel to these experimental developments, progress has been made with electromagnetic bistatic models [25,26,27,28,29], improving scientific understanding of the scattering effects taking place in GNSS reflectometry. One important result is the hypothesis proposed by [9] and experimentally confirmed by [30], according to which the combined measurement of Left Hand Circular Polarization (LHCP) and Right Hand Circular Polarization (RHCP) reflected signals can improve the retrieval of soil moisture content. This is possible because the ratio of these two signals is sensitive to soil moisture, while being more robust with respect to surface roughness effects.

Over the last ten years, GNSS-R instruments with polarimetric capabilities have been developed, and have demonstrated their ability to monitor land surfaces in both LHCP and RHCP. The SAM receiver [11] was used in several campaigns [30], with its main limitation being the use of only one reflected channel, resulting in its inability to measure both polarizations simultaneously. More recently, the PYCARO instrument was modified in order to simultaneously retrieve both polarizations [23], but little information is available concerning this receiver and its performance. Finally, a 4-channel lightweight instrument was proposed for measurements made by Unmanned Aerial Vehicles (UAVs) [31]. Its main limitations arise from (1) the use of different components in the receiving channels, leading to power and phase variations between the LHCP and RHCP channels, and (2) the instrument’s antennas, which are affected by cross-polarization isolation issues, thus preventing reliable polarimetric measurements. In the present paper, an airborne polarimetric GNSS-R instrument, referred to as GLObal navigation satellite system reflectometry Instrument (GLORI) is presented. The main geophysical land surface parameters of interest in this study are: soil moisture, vegetation water content, forest biomass and inland water body altimetry. This instrument has been installed as a permanent payload on a French ATR-42 research aircraft, allowing both direct and reflected LHCP and RHCP GNSS signals to be collected simultaneously during flights. Section 2 of this paper provides a detailed description of the GLORI instrument, and focuses on the system’s polarimetric performance and cross-channel calibration, demonstrating that it is well suited to applications requiring polarimetric GNSS-R measurements. Section 3 describes the airborne campaigns carried out with the GLORI instrument, designed to measure the instrument’s sensitivity to various geophysical parameters. Section 4 describes the data processing techniques used to analyse the recorded signals. Initial results and the instrument’s data quality are discussed in Section 5. Our conclusions on the instrument’s suitability to GNSS-R land measurements are presented in Section 6.

## 2. The GLORI GNSS Reflectometry Instrument

### 2.1. General System Description

The GLORI instrument is derived from the cGNSS-R family [32]. It is a highly versatile, low-cost, 4-channel GNSS-R receiver, built using mainly commercial off-the-shelf components. Direct and reflected GNSS signals are received by two hemispherical GPS dual-frequency (L1 and L2) dual-polarization active antennas: the Zenith RHCP antenna, hereafter referred to as ZR, mounted on the upper part of the aircraft fuselage, and the Nadir dual polarization (LHCP and RHCP) antenna, hereafter referred to as NL and NR, mounted on the lower fuselage of the aircraft. One of the main advantages of the instrument is its ability to simultaneously record both LHCP and RHCP polarizations, allowing it to analyse depolarization effects produced by the land surface.

The received signals are filtered to reduce out-of-band noise, which could saturate the front-end. Channel cross-calibrations are performed during each flight, in order to assess the channel-to-channel gain imbalance possibly caused by differences in cable losses or digitizer amplification.

The RF signals are fed into three of the four available channels of the L-band front-end, which ensures direct in-phase and quadrature (IQ) down-conversion, 2-bit signal decimation, and serialization to a USB 2.0 interface.

**Figure 1:**
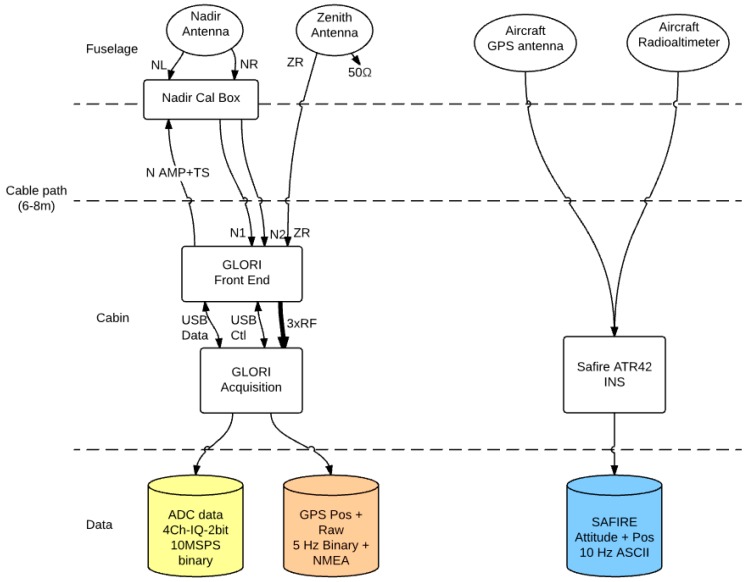
Block diagram of the GLORI instrument.

A standard PC is used to acquire the raw data signals. Signal acquisition, tracking and observable computations are performed during post-processing, following the flights. The main technical specifications of the instrument are presented in Table 1, and an instrument block diagram is shown in Figure 1.

### 2.2. Instrument Design Details

The GLORI instrument comprises three subsystems: two antennas, the front-end receiver, and the acquisition unit.

#### 2.2.1. Antennas

**Figure 2:**
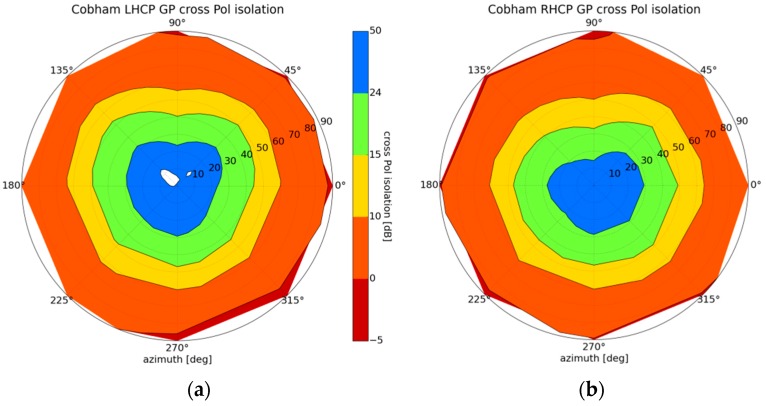
Nadir-looking antenna (Cobham DS1563) cross-polarization isolation: (**a**) LHCP Port; (**b**) RHCP Port. The radial scale is the incidence angle. Measurements performed at the Centre National d’Etudes Spatiales (CNES), Toulouse France.

The instrument is currently fitted with dual polarization (one output per polarization) GPS L1/L2 antennas. The zenith-pointing antenna is active (Antcom 42G1215RL-AA-XT-1 [33]), and was selected for its form factor and compliance with aeronautical standards. On this antenna, only the RHCP polarization (ZR) is used. The nadir antenna is passive (Cobham DS1563 [34]), and both polarizations (Nadir LHCP (NL) and Nadir RHCP (NR)) are connected to a transfer switch, followed by Low Noise Amplifiers (LNAs) with a noise figure <1 dB. The Nadir antenna was selected for its cross-polarization isolation performance, and because it allows a more sensitive LNA to be used than in the case of the majority of active antennas. Figure 2 shows the cross-polarization isolation for both polarization ports. The blue colouring corresponds to an antenna cross polarization isolation better than 24 dB (GPS transmit antenna specifications): this is observed for angles up to 30° from boresight (60° elevation) at the LHCP port, and up to 25° (65° elevation) from boresight at the RHCP port. The green colouring shows that the cross-polarization isolation is better than 15 dB at angles up to 45° from boresight (45° elevation), for both LHCP and RHCP ports. A cross-polarization performance of less than 15 dB (yellow and red colouring), is considered inadequate for the purpose of polarimetric measurements [30]. The antennas are characterised by a small defect at 90° azimuth, reducing the cross-polarization isolation by approximately 10 dB, which is taken into account by filtering the measurements recorded with this portion of the antenna.

#### 2.2.2. Front End

**Figure 3:**
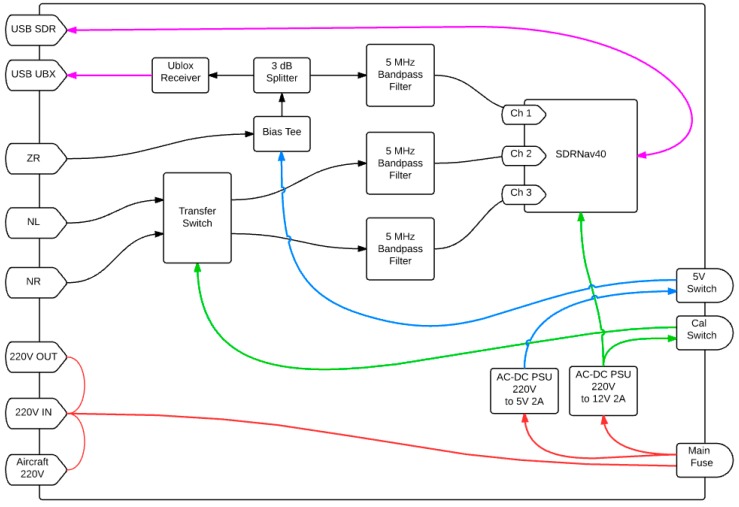
GLORI Front End. Black: RF signal; Red: 220 V, 50 Hz power supply; Blue: 5 V Power supply; Green: 12 V Power supply; Purple: USB Serial Data Link.

The GLORI front-end (Figure 3) filters and amplifies the received signals, and ensures channel switching for calibration purposes, as well as baseband mixing and D/A conversion through the use of a SDRNav40 v2 receiver [35]. The latter is a multi-frequency, quad-channel, tuneable receiver using synchronized MAX2112 tuners and MAX19505 A/D Converters, which ensure direct down conversion and sampling of the signals.

#### 2.2.3. Acquisition Unit

The acquisition unit is an industrial PC running Windows 7, with 8 GB of RAM and an Intel core i7 processor. The system hard drive is a single Solid State Drive (SSD), whereas the data hard drive consists of two 1 TB SSDs, allowing more than 20 h of continuous raw data to be recorded at the nominal 10 Mbps sampling frequency.

#### 2.2.4. Auxiliary sensors

As shown in Figure 1, the following ancillary data are measured and recorded:

1. The aircraft’s distance from the ground, using a 4.2–4.4 GHz Thomson ERT 900 Radio Altimeter with an accuracy of approximately 2%.
2. Attitude and position information using the aircraft’s integrated Inertial Navigation System (Ixblue AirINS [36]) (GPS time, GPS altitude, latitude and longitude, heading, pitch, roll, and 3-axis speed).
3. GPS Position derived from a Ublox Neo6T receiver [37] connected to the Zenith RHCP antenna, which records the aircraft’s position and GPS information (relative elevation and azimuth, code phase, Doppler frequency, signal to noise ratio, pseudo-range) at a frequency of 5 Hz.

### 2.3. Instrumental Performance

#### Channel Cross-Calibration

In the case of polarimetric measurements, gain discrepancies between the Nadir LHCP and RHCP paths can lead to significant errors. In order to estimate the relative power responses and possible variations of the GLORI acquisition channels, a calibration routine was performed before and after each flight, while the aircraft was on the ground. For this purpose, the Zenith RHCP antenna (ZR) is sequentially connected for short 16-s sequences to each of the GLORI ports (1, 2 and 3), by means of transfer switches (Table 2).

**Figure 4:**
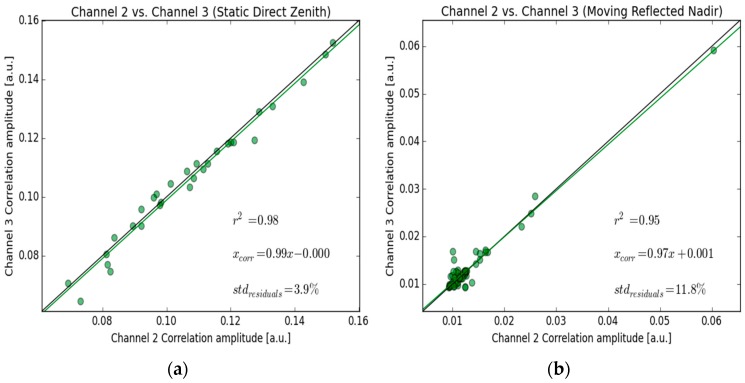
Cross-calibration results for the entire campaign. (**a**) Using the Zenith RHCP signal as a reference from the ground; (**b**) Using the Nadir LHCP signal while flying over inland water. The green line represents the calibration line between the two channels. The green dots represent the individual observations (one per calibration sequence for each observed satellite). The black line is used to indicate the response for an ideal calibration curve (Channel 1 = Channel 2). *r*^2^ is the squared Pearson coefficient.

The instrument’s relative polarimetric calibration factor is then computed from the ratio of the Zenith RHCP correlation power measured in Channel 2 (Cal_2), to the Zenith RHCP correlation power measured in Channel 3 (Cal_3). The calibration factor is defined as the slope of the linear regression adjusted to measured values in each channel. The inter-channel error is estimated as the standard deviation of the residuals, expressed as a percentage. A system with perfectly equivalent responses in both channels would have a calibration factor of 1, and an inter-channel error of 0 (0 dB), represented by the black line in Figure 4.

Figure 4a shows the results of the polarimetric calibration (between Channels 2 and 3) for the entire campaign. The correlation between different pairs of channels is found to be very good (coefficient of determination *r*^2^ = 0.98), with a calibration factor of 0.99 (almost identical power in each channel). The standard deviation of the calibration data residuals, which is likely to be the consequence of apparent movements of the GNSS satellites with respect to the antenna’s radiation diagram, is of the order of 4%, leading to an inter-channel calibration error of approximately 0.35 dB.

Another more experimental approach, similar to that described above, was also tested: this technique uses a Nadir LHCP signal reflected by a water surface as the reference signal. In this case, the power is sufficiently strong to track reflected satellite signals. The data generated by the calibration stages, Cal_1 and Cal_3, can then be used to compute the polarimetric calibration factor. In this case, the inter-channel error increases to approximately 12% (1 dB). This is probably caused by the aircraft’s imperfect trajectory, combined with roughness effects over the water surfaces (Figure 4b). The former method was used to estimate the instrument’s polarimetric accuracy, which was found to be better than 0.35 dB.

## 3. The GLORI Campaigns

### 3.1. Overview

Two campaigns were carried out in 2014 and 2015:

- The GLORI 2014 campaign was a flight of opportunity, originally planned for the validation of KuROS, a Ku band Doppler scatterometer developed in preparation for the CFOSAT satellite mission [9]. The flight plan was not optimized for reflectometry studies, nor for the collection of ground truth data. The aim of this campaign was to test the instrument’s in-flight performance.
- The GLORI 2015 campaign lasted for a period of three weeks, and was dedicated to GNSS reflectometry over land, associated with simultaneous, collocated ground-truth measurements.

### 3.2. GLORI 2014 Campaign

**Figure 5:**
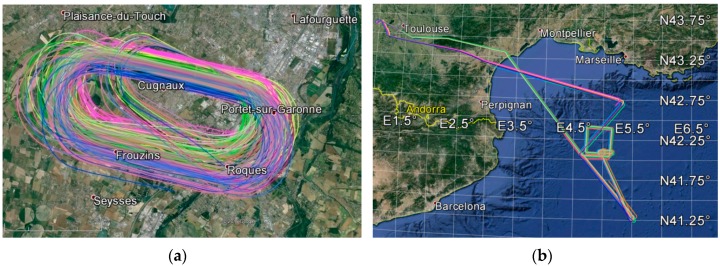
Racetrack flight over Francazal Airport (**a**) and Test Flight over the Gulf of Lion (**b**). The coloured lines represent distinct satellite reflection tracks (GLORI 2014 Campaign).

In the case of the GLORI 2014 campaign (Figure 5), three flights were made, with continuous GNSS-R measurements (5 h of polarimetric raw data):

- Two racetrack-type flights were made over the Francazal airport area: Flights 2014-39, (44 min of recorded data) and 2014-41, (31 min of recorded data), at a constant altitude of approximately 450 m above the surface.
- Flight 2014-40 was made over the Gulf of Lyon, during which 3 h and 43 min of data were recorded, in order to calibrate the KuROS scatterometer [38] at two different altitudes: 2000 m and 3000 m.

No ground-truth data were collected during these flights, and the sea was considered to be calm during flight 2014-40.

### 3.3. GLORI 2015 Campaign

#### 3.3.1. Flight Plan

**Figure 6:**
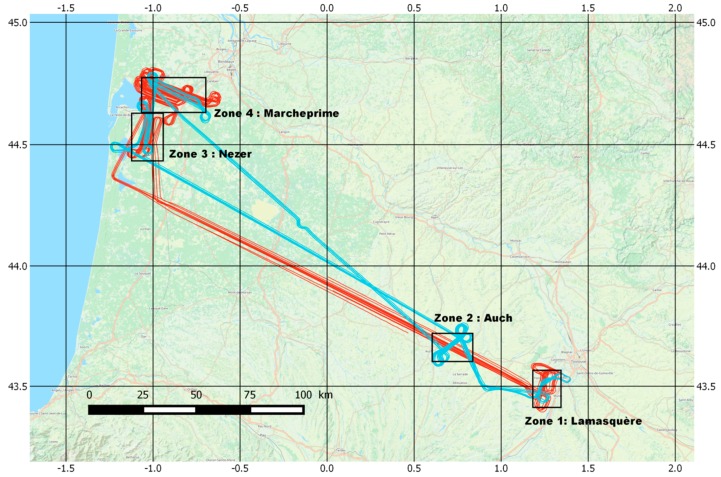
Location of the GLORI 2015 Test Sites in Southwest France. The blue lines show an example of reflection tracks from a Type A (short) flight, whereas the red lines correspond to a typical Type B (long) flight.

The 2015 GLORI campaign was carried out in Southwest France and was designed to assess the feasibility of using GNSS-R for the retrieval of land surface parameters: soil moisture (SM), soil roughness (SR) and aerial biomass of agricultural and forest vegetation. In order to comply with airport flyover regulations, two types of flight were planned and carried out (Figure 6):

- *Type A flights (short)*: ~2 h and 15 min daytime flights, focusing mainly on agricultural areas.
- *Type B flights (long)*: ~3 h 15 min night flights, focusing mainly on forest-covered areas.

During the course of each flight, large lakes were overflown to permit the calibration of reflected channels, and to assess the capabilities of GNSS-R altimetry over inland water bodies. In addition to a high percentage of straight horizontal flight transects, wing wags with a ±25° roll over land and water, as well as circular flights, were carried out. These movements were useful for the analysis of the antenna radiation pattern, and also contributed to a better understanding of the physical signals acquired by the GNSS instrument. In total, more than 15 h of raw data were recorded over various surfaces.

#### 3.3.2. Validation Sites

In the case of the 2015 campaign, four different test sites were selected for the acquisition of GLORI data over land. The map in Figure 6 shows the location of the overflown zones, whereas Table 3 summarises the main characteristics of these test sites.

In parallel with the 2015 GLORI GNSS-R airborne acquisitions, various *in situ* measurements were made on these sites (vegetation biomass, roughness, soil moisture, and leaf area index), in order to validate the GLORI data, to improve the direct bi-static models, and finally to optimise the inversion algorithms.

Zone 1 and Zone 2 correspond to agricultural areas with a temperate climate. *In situ* measurements were carried out simultaneously with the airborne measurements, on more than 20 agricultural fields characterised by different types of land use (bare soil, cereals, corn, soybean, *etc.*). These measurements concerned soil moisture (performed with Theta probes) and roughness (estimated using a needle profilometer), as well as a description of the vegetation cover (Leaf Area Index or LAI from hemispheric photography, water content, vegetation height). Straight-line flight transects covered all of these test fields.

The Zone 3 and Zone 4 sites are located in the Landes forest, where maritime pine stands surround a small number of very large agricultural fields. In the case of the Zone 4 site, the airborne GNSS-R measurements involved 11 transects covering an area of more than 500 km^2^. In this zone, 11 fields with various crops (corn, potato, carrot, bean) were sampled. Soil moisture and various characteristics of the vegetation cover (LAI, cover fraction, vegetation height) were measured during each flight. In both zones, more than 100 test plots were selected in forest stands. The age of the trees of each sampled stand was estimated from field observations or given by the forest manager. Diameter at breast height, tree total height, and density (number of trees per ha) were measured to estimate the biomass from allometric equations [39,40,41]. It is interesting to note that the trunk dry biomass in the Landes forest can reach as much as ~150 t/ha [42], corresponding to an above ground biomass of about 200 t/ha [39], allowing the study of a rather large range of biomass densities. Complementary ground truth measurements were carried out during the flights, in order to evaluate the soil moisture, the LAI, and the vegetation cover fraction of the undergrowth.

## 4. GLORI Airborne Data Processing

### 4.1. Observed Variables

The proposed data processing algorithms (see Section 4.2) are used to compute different products, for the scientific analysis of the test zones. The direct and reflected GNSS signals, *u_d_* and *u_r_*, are cross-correlated with pseudo-random noise (PRN) code replicas *a*(*t*) shifted by various multiples of a time-domain interval *τ*, and a Doppler frequency interval *f*. The resulting correlation can be expressed as:

(1) $$Y_{r}\left(\tau ,f\right)=\frac{1}{T_{i}}\int_{T_{i}}u_{r}a\left(t-\tau \right)e^{i\left(f_{c}+f\right)t}dt$$

where *T_i_* is the coherent integration time, during which the delay and Doppler parameters are kept constant, and *f_c_* is the frequency of the downconverted incoming signal.

As a result of the scattering processes experienced by the signals reflected by the Earth’s surface, the correlation peak derived from a short coherent integration is difficult to interpret. Incoherent integration is thus required to reduce speckle noise in the reflected signals [43]. It can be shown that the mean power of the incoherently averaged correlation *Y_r_*(*τ,f*) given by Equation (1), known as the Delay-Doppler Map (DDM), and for incoherent scattering it can be expressed as [44]:

(2) $$\langle {\left|Y_{r}\left(\tau ,f\right)\right|}^{2}\rangle =\frac{T_{i}^{2}PtGt\lambda^{2}}{{\left(4\pi \right)}^{3}}\iint_{A}\frac{Gr\left(\vec{\rho }\right)}{R_{0}^{2}\left(\vec{\rho }\right)R^{2}\left(\vec{\rho }\right)}\sigma_{pq}^{0}\chi^{2}\left(\vec{\rho },\delta \tau ,\delta f\right)d\vec{\rho }$$

where *Pt* and *Gt* represent the satellite transmit power and antenna gain, respectively, *λ* is the signal wavelength (19 cm at the GPS L1 frequency), $Gr\left(\vec{\rho }\right)$ is the receiving antenna gain, $R_{0}\left(\vec{\rho }\right)$ and $R\left(\vec{\rho }\right)$ are the distances between the specular point and the transmitter and receiver, respectively, $\sigma_{pq}^{0}$ is the polarization-dependent, bi-static radar coefficient, and *χ*, known in radar terminology as the Woodward ambiguity function (WAF), accounts for the signal modulation characteristics. The integration domain A is referred to as “the glistening zone” which is the active scattering area that effectively contributes to the reflected signal. The DDM $\langle {\left|Y_{r}\left(\tau ,f\right)\right|}^{2}\rangle$ corresponds to the GNSS power scattered by the surface, as a function of delay and Doppler frequency. Due to the spatial filtering of the WAF, the DDM points can be related to contributions from cells on the surface which correspond to the intersection of iso-delay and iso-Doppler lines.

For a scattering surface with a small roughness in comparison to the incident wavelength, the coherent scattering component is the predominant one, and therefore most of the power scattered from the surface will come from the vicinity of the specular point. In practice, this translates to a DDM in which most of the energy is concentrated around a single Delay-Doppler bin corresponding to the specular point position. In this case, the measured reflected power originates from a limited area on the ground called the first Fresnel zone [45], and not from the whole glistening zone as defined above. The scattering from a rough surface is incoherent, which has a more isotropic nature that the coherent component. This results in a DDM with energy spread over several delay-Doppler bins originates from surfaces with stronger incoherent scattering, *i.e.*, greater surface roughness. DDMs from various types of land surface are presented and commented in Section 5.1.

**Figure 7:**
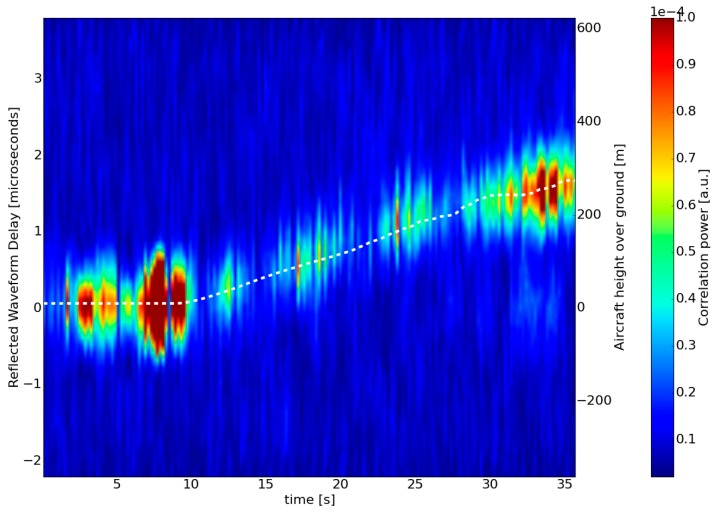
Variation of reflected correlation waveforms Y_r_ (arbitrary units) *vs.* time during the take-off sequence (29 June 2015, 5:33:41 UTC, PRN13, incoherent integration time 200 ms). The aircraft’s height above ground, provided by the radio altimeter, is shown by the white dashed line.

Figure 7 illustrates the time series (*x*-axis) of reflected waveforms, and is defined as the correlation power (coloured scale, arbitrary correlation units) as a function of time-domain delay (*y*-axis), at a constant Doppler frequency. The data are derived from GPS PRN 13, recorded during the aircraft’s take-off from Toulouse Francazal airport on 29 June 2015. The waveform’s maximum clearly shifts towards longer delays as a function of time. This shift corresponds to the aircraft’s gain in altitude during take-off (superimposed white dashed line), leading to an increase in the delay between the direct and reflected signals.

The ratio between the peak time series of the reflected and direct amplitude waveforms is known as the Interferometric Complex Field (ICF) [46]. In the case of systems with identical zenith- and nadir-pointing antennas aligned with the ground (platform tilt and roll angles equals to 0°), this ratio is used to compensate for propagation noise, clock drift errors, Doppler tracking errors, *etc.*, and provides a product suitable for e.g., phase altimetry.

In the case of GLORI, we produced a generic form of the ICF, which takes into account the differences in noise and antenna gain between the direct and reflected channels. By dividing the peak value of the corrected reflection waveform by the peak value of the corrected direct waveform, the corrected Interferometric Complex Field *ICF_corr_* can be computed as:

(3) $$ICF_{corr}=\frac{\left|Y_{r,\text{max}}\right|-B_{r}}{\left|Y_{d,\text{max}}\right|-B_{d}}e^{j(\phi_{r,\text{max}}-\phi_{d,\text{max}})}\frac{G_{d}}{G_{r}}$$

where *B_r_* and *B_d_* are the waveform noise floor levels, and *G_r_* and *G_d_* are the azimuth- and elevation-dependent antenna gains, *φ_r_*_,max_ and *φ_d_*_,max_ are the GPS carrier phases of the direct waveform peaks, and |*Y_r_*_,max_| and |*Y_d_*_,max_| are the moduli of the reflected and direct waveform peaks, respectively. *ICF_corr_* is computed for both RHCP and LHCP reflected polarizations (direct polarization being fixed as RHCP), leading to respectively the co-polarized *ICF_corr.rr_* and cross polarized *ICF_corr,rl_*.

The apparent reflectivity is the main product of interest in GNSS-R reflectometry for land surfaces. It is related to the relative power reflected coherently from the ground [13] in a given polarization. For a polarization *pq*, the apparent reflectivity Γ_*pq*_ can be expressed as the squared modulus of the coherently averaged ratio of the reflected *Y_r,q_*(*τ,f*) to direct waveforms *Y_d,p_*(*τ,f*). By assuming *f* to be aligned with the Doppler frequency shift of the direct signal, the apparent surface reflectivity Γ’_*pq*_ can be written as in [13]:

(4) $$\Gamma_{pq}^{\prime }={\left|\langle \frac{Y_{r,q}\left(\Delta \tau ,f\right)}{Y_{d,p}\left(0,f\right)}\rangle \right|}^{2}$$

where Δ*τ* represents the difference in delay between the direct and reflected paths, and is computed first by a coarse geometrical approximation from altitude and angle, and then by looking for the maximum of the reflected signal.

However, for surfaces with a strong incoherent scattering component, the phase of the reflected signal changes in a random way, preventing the coherent average of the signal. For this reason, the apparent reflectivity Γ’_*pq*_ needs to be computed in a way that allows for the separation of coherent and incoherent components. It was show that this can be done from the ICF, using the method described in [13]:

(5) $$\Gamma_{pq}^{\prime }=\langle {\left|ICF_{corr,pq}\right|}^{2}\rangle -\sigma_{\left|ICF_{corr,pq}\right|}^{2}$$

where ${\sigma^{2}}_{\left|ICF_{corr,pq}\right|}$ is the variance of the ICF over the incoherent averaging period.

Finally, the observable called “polarimetric ratio” defined as Γ_*pq*_/Γ_*rr*_ is computed. It is considered as being a stable indicator of soil moisture content for terrains with limited roughness (standard deviation < 3 cm) [13].

### 4.2. Data Processing Implementation

**Figure 8:**
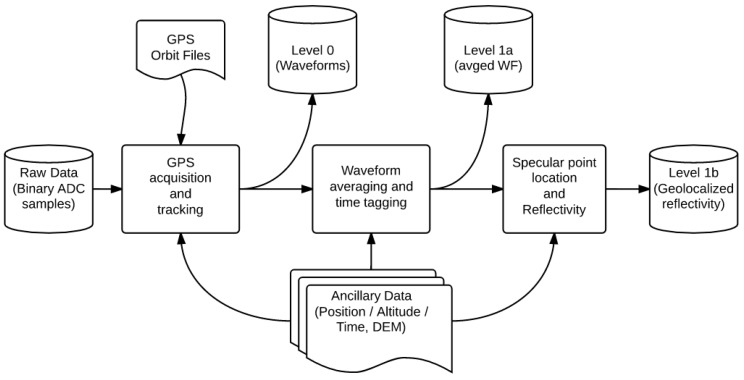
Processing Block Diagram.

The raw data are processed in several steps, which currently lead to the generation of three different data products (Figure 8):

1 GPS acquisition and tracking computations are performed on the raw, direct signal (zenith-pointing antenna), whereas the reflected signals (nadir-pointing antenna) are processed in a master-slave scheme using tracking parameters from the direct signal. This processing step leads to the generation of Level 0 files, consisting in time series of 5 ms coherently integrated, complex, direct and reflected (LHCP and RHCP) waveforms. Following various empirical tests, a coherent averaging time equal to 5 ms was selected. This value provides a good compromise between thermal noise reduction, and the conservation of incoherent signals, over forested areas in particular. However, other values of integration time could be used for specific studies in the future. In addition to the GNSS-R data, metadata and decoded GPS message-related information corresponding to each raw file of 30~60 s duration were stored. Optionally, the DDM can be computed using the method described in [21].
2 In a second step, the relevant ancillary data, including aircraft attitude (yaw, pitch and roll), transmitter elevation and azimuth, are interpolated over the integration step, and calibration and correction factors are computed (antenna radiation pattern, inter-channel calibration). The waveforms are precisely time tagged from the GPS message, the waveform maxima are detected, and the corrected ICFs are computed for each polarization and coherent integration time step. Then, the ICFs are incoherently averaged (200 ms) and their standard deviation is estimated in order to be able to compute apparent reflectivity.
3 For each flight, the data are then aggregated, including ancillary and calibration information. During this step, the apparent reflectivity is calculated, as well as the location and shape of each specular ellipse corresponding to the first Fresnel zone [45] from the ancillary data and precise time tagging of the ICFs.

## 5. Preliminary Results and Discussion

In this section, some very preliminary results of the experimental campaign and of the quality of the GLORI measurements are illustrated over the forest (Zone 3) and the agricultural (Zone 4) areas in Les Landes region.

### 5.1. LHCP DDM Analysis

**Figure 9:**
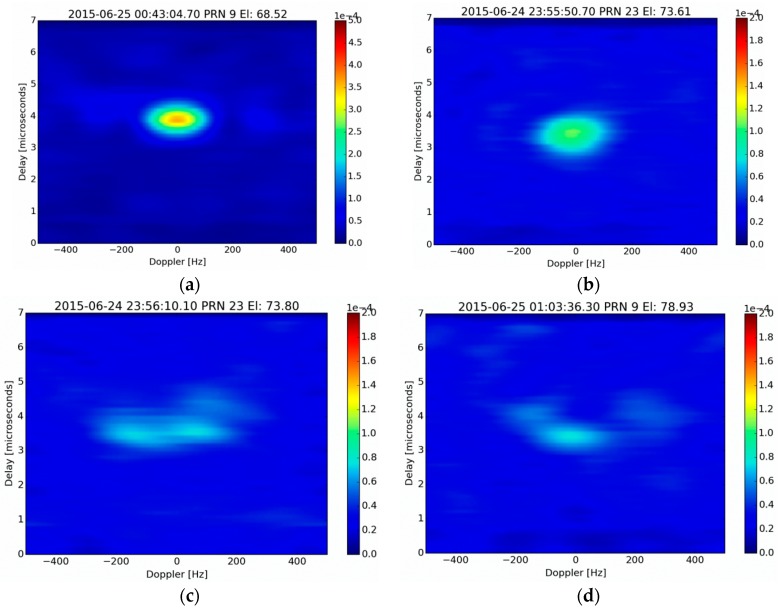
Delay Doppler Maps of reflected signals over: (**a**) water (**b**) a carrot field; (**c**) a mature corn field (**d**) a pine-tree stand (age approx. 15 years). Altitude of the aircraft: approximately 600 m, coherent integration time: 5 ms, incoherent averaging time: 200 ms. The color scale indicates the correlation power in arbitrary units. A different scale is used in the plot (**a**) to avoid saturation.

Figure 9 provides four examples of DDMs generated over different types of surface, from an altitude of approximately 600 m, with a coherent integration time set to 5 ms and an incoherent averaging time set to 200 ms. Figure 9a shows the DDM generated from a pond flyover. It can be seen that all of the reflected energy is concentrated in the zero Doppler bin, showing strong signal coherency, as could be expected from a reflective surface such as calm water. Figure 9b shows the DDM produced over a recently planted carrot field, with very little vegetation cover. It can be seen that the energy reflected by the surface is also concentrated in the zero-Doppler bin, thus showing that it produces a strongly coherent reflection, although the reflected energy is 10 times weaker than that measured over water. Figure 9c shows the DDM produced over a mature corn field. It seems that most of the reflected energy also originates from a limited Delay-Doppler area, even if a spread in the Doppler range seems to indicate a somehow stronger incoherent scattering contribution, which is probably caused by reflected signal interactions within the vegetation layer. Finally, Figure 9d shows the DDM produced over a 15 year-old pine forest, in which a part of the reflected energy also seems to be spread over several Doppler bins. It is likely this result is also related to incoherent scattering of the reflected signals from the forest canopy.

These DDMs were measured from low altitude flights (small collection area), with a short incoherent averaging time period. The use of longer incoherent averaging time periods over larger homogenous fields would allow to better characterize the signal structure by reducing the speckle noise and could be used for a possible estimation of coherent and incoherent scattering contribution based on an analysis of the DDMs. Moreover, improved electromagnetic modelling of such processes will be needed, in order to gain a more detailed understanding of the mechanisms involved in the structure of the reflected signals, in particular those produced over vegetated surfaces.

### 5.2. Reflectivity Maps

In this section, the instrument’s overall stability and sensitivity to various terrain types is estimated by mapping the reflected power over the full study area.

**Figure 10:**
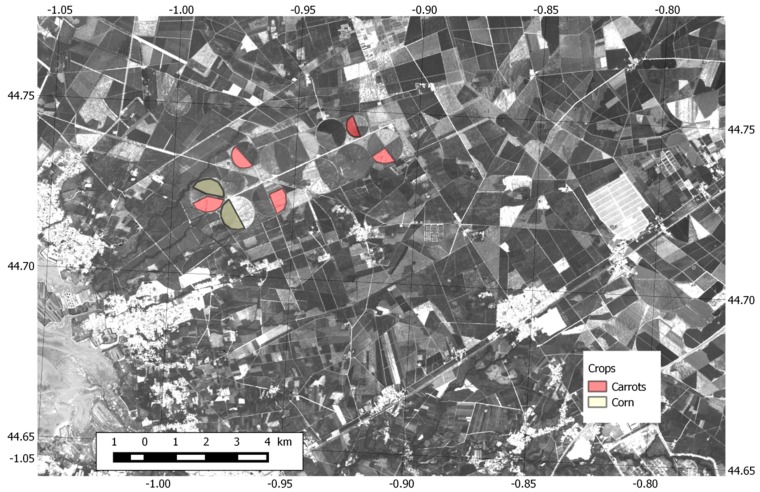
Reference Landsat 8 reflectance image recorded on 30 June 2015 (Band 8, Panchromatic, 15 m resolution) over Zone 4. The white rectangle indicates the agricultural area of interest, with several circular fields. Corn plots are highlighted in brown color, and carrot plots are highlighted in pink color.

Figure 10 shows a reference image over the Zone 4 recorded on 30 June 2015 by Landsat 8 in the panchromatic channel (15 m spatial resolution). In this image, among other land uses, in particular maritime pine forest, circular agricultural fields can be seen near the image center (indicated by a white rectangle). Their cover fraction is relatively well correlated with the reflectance in the visible: white areas correspond to bare soil, black areas correspond to dense vegetation.

**Figure 11:**
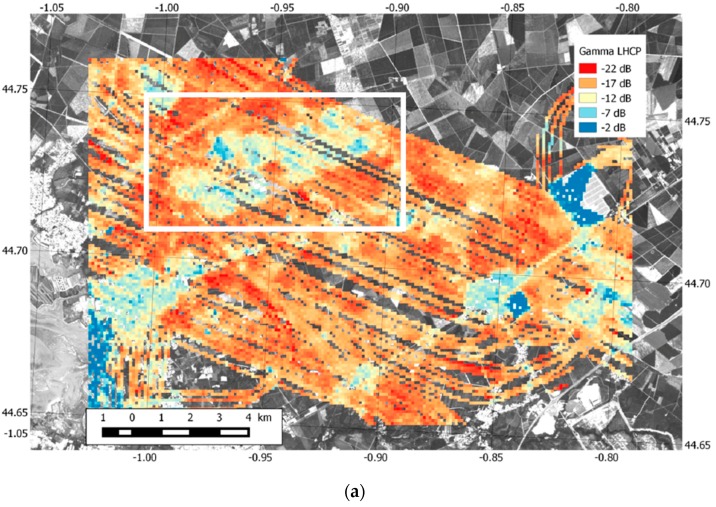

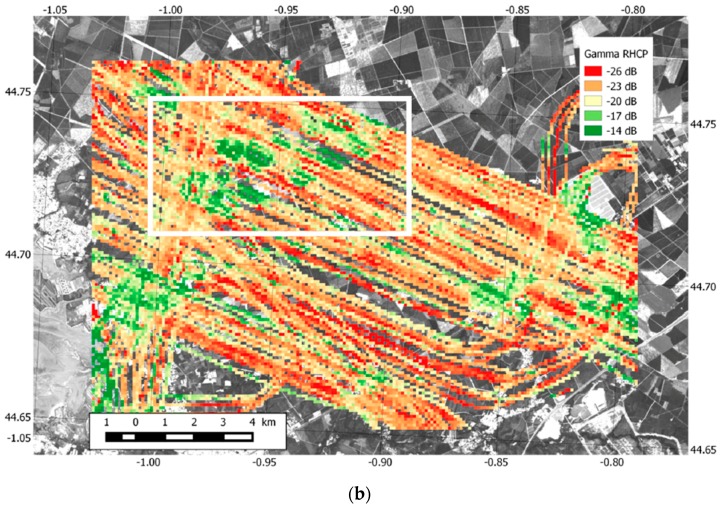
(**a**) GLORI LHCP reflectivity measurements recorded during the 2015 airborne campaign over Zone 4, at elevations varying between 45° and 90°. The data are averaged over 100 × 100 m^2^ pixels; (**b**) GLORI RHCP reflectivity measurements under the same conditions. Red color means weak microwave reflections, while blue and green colors mean strong microwave reflections.

Figure 11 provides an overall view of the ground reflectivity measured in Zone 4 (Figure 6) by the GLORI instrument. By averaging the measurements recorded during all flights, taken from a large range of elevation angles (between 45° and 90°), it was possible to reconstruct a nearly complete image of this zone (10 × 15 km^2^), with a resolution of 100 m.

Figure 11a shows the reflectivities measured using LHCP polarization. The dynamic range (~20 DB) of the recorded signals (from −22 to −2 dB) is represented here by the coloured pixels. The main features of this area are clearly visible: roads, urban areas, water, a solar power plant, pine stands and an agricultural area. In the agricultural area (white rectangle), the bare soil fields have a stronger reflectivity signature than the zones covered by vegetation. The weakest reflectivity signatures can be observed for the closed-canopy forest. One remarkable feature of the LHCP measurements is the homogeneity of the recorded signals, despite the use of measurements taken from a wide range of elevation angles: as predicted by the models, the LHCP signal is only weakly dependent on elevation angle, when this latter is greater than 45° [30].

Figure 11b shows the corresponding map for the RHCP polarization. The overall dynamic range of the signal is approximately 12 dB (from −26 to −14 dB). This map reveals that the RHCP signals have a much stronger dependence on incidence angle, creating stripes along the flight tracks. This outcome is predicted by the models, with the signal intensity increasing with decreasing elevation angles, for all types of terrain. Although the RHCP and LHCP signals are sensitive to the same surface areas, a more detailed analysis of their dependence on elevation angle would be needed, to determine the exact influence of the terrain characteristics. Overall, the GLORI instrument has a good dynamic range (at least 20 dB) and the ability to characterize various surface types. The observed relationship between the measured instrument’s reflectivity and elevation angle is as expected, with the power remaining stable in LHCP, and increasing with decreasing elevation angles in RHCP.

### 5.3. Apparent Reflectivity Analysis: Agricultural Areas

In this section, we study the influence of two different kinds of agricultural crops on the apparent surface reflectivity and on the circular polarisation ratio of the reflected signals. We analysed two main types of crops: (i) recently planted carrot fields, which can be considered as equivalent to bare soil fields since the carrot plants are small and do not completely cover the ground, with a measured height ranging from ~0 to 25 cm; and (ii) more mature corn crops with an higher cover fraction, and a plant height varying between 50 and 150 cm. In both cases the field soil moisture was relatively stable (estimated with theta probes as ranging from 0.1 to 0.15 m^3^/m^3^). The location of the plots can be seen in Figure 10.

**Figure 12:**
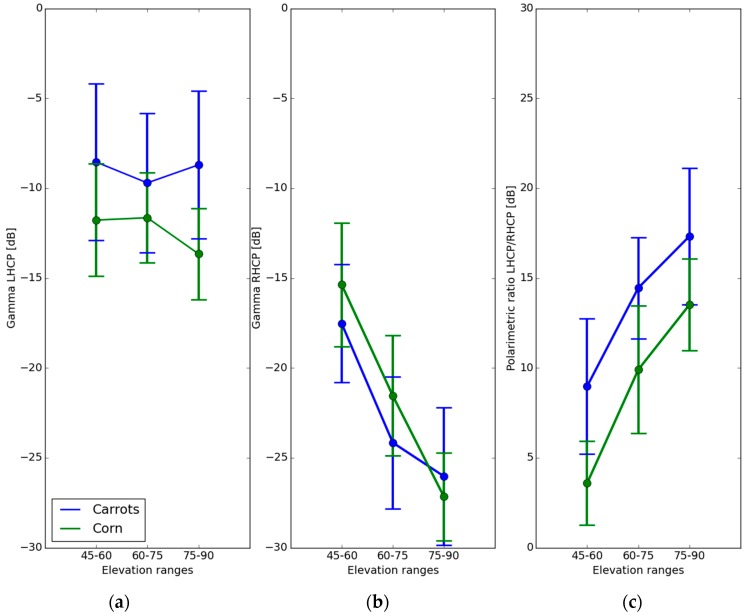
Average apparent reflectivity and polarimetric ratio for 2 classes of crops (recently planted carrots, considered to be equivalent to bare fields, and mature corn), and 3 classes of elevation (45°–60°, 60°–75° and 75°–90°). (**a**) apparent LHCP reflectivity; (**b**) apparent RHCP reflectivity; (**c**) LHCP/RHCP polarisation ratio. Vertical error bars represent the 1-sigma standard deviation.

Figure 12a shows the averaged apparent LHCP reflectivity for these two types of cover and three ranges of elevation angle (45°–60°, 60°–75°, 75°–90°). In the case of the carrot field, the apparent reflectivity varies between −8.5 and −9.5 dB, depending on the range of elevation angles, and in the case of the corn field, it varies between −11.5 dB at elevation angles below 75°, and −13.5 dB at angles greater than 75°. This change in reflectivity is consistent with the trend observed under conditions of increasing Plant Water Content (PWC), related to increasing plant biomass [11].

In Figure 12b plots the averaged apparent reflectivity for the RHCP polarization under the same conditions. It can be seen that the reflectivity decreases strongly with increasing elevation angle, for both types of crop. For elevations varying between 45° and 75°, the cornfield reflectivity is approximately 2 dB higher than that of the carrot field, whereas it is slightly lower at elevations between 75° and 90°. This behaviour will be analysed in more detail in future studies. Generally, the RHCP reflectivity levels are lower than the LHCP ones for the studied conditions. The 1-sigma standard deviation of the measurements is on the order of 2.5 to 4 dB, as illustrated by the vertical error bars in Figure 12.

Figure 12c, plots the average LHCP/RHCP polarization ratio for both types of crop. It can be seen that this ratio increases with increasing elevation angle, with the carrot field reflectivity being approximately 4 dB higher than that of corn fields, at all elevation angles. Thus, it seems that the GLORI observations have very distinct signatures over the two types of agricultural cover. Further studies and detailed models will be needed, in order to establish a physical interpretation of these measured trends.

### 5.4. Apparent Reflectivity Analysis: Forests

In this section, we assess the instrument’s sensitivity to various forest conditions over the pine forests of Zone 3 (Nezer area). This area was well documented and the exact age of all stands was known from forest management data. Although a stand’s age is not linearly correlated with its biomass, it can be considered as a reasonably good proxy of it.

**Figure 13:**
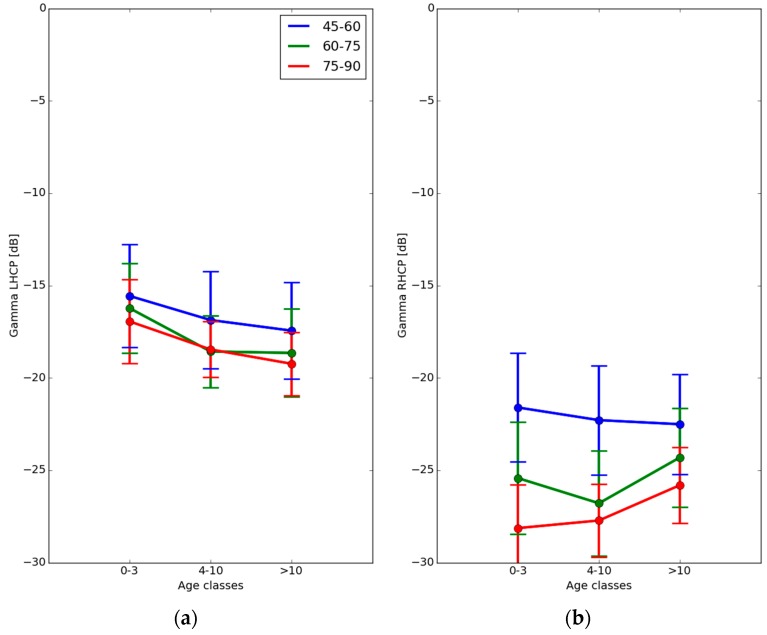
Average apparent reflectivity for various forest plots grouped by age category, for three classes of elevation angle. (**a**) LHCP observations, (**b**) RHCP observations. Vertical error bars represent the 1-sigma standard deviation.

Figure 13 shows the average value of the apparent LHCP and RHCP reflectivities, for three elevation angle ranges (45°–60°, 60°–75°, 75°–90°), and for three classes of forest age: very young (0–3 years), medium (4–10 years) and closed canopy (>10 years).

At all elevations, the apparent LHCP reflectivity decreases by approximately 2 dB, from −16.5 to −18.5 dB, with increasing forest age. This is in agreement with measurements reported for forest stands <200 t/ha in a previous study [13]. The LHCP reflectivity is also weakly dependent on incidence angle, with a variation of approximately 1.5 dB between high and low elevation angles, as has also been reported by Egido *et al.* [13].

In the case of RHCP polarization, the apparent reflectivity has a more complex dependence on forest age, as shown in Figure 13. This behaviour needs to be discussed in more detail, through the use of a numerical scattering model able to simulate the individual reflectivity contributions by the soil, the trunks, branches, and leaves of the forest canopy [47]. The 1-sigma standard deviation of the measurements is on the order of 1.5 to 3 dB, as illustrated by the vertical error bars in Figure 13.

The GLORI instrument is thus sensitive to changes in forest biomass, and can detect apparent reflectivities down to approximately −30 dB. As reported by [47,48], this sensitivity should be sufficient for the characterisation of forests having an above ground biomass up to 200 t/ha such as the ones composed of maritime pines, but not applicable for denser forests where the above ground biomass can go up to 600 t/ha, such as in tropical regions [49].

## 6. Conclusions

The GLORI instrument was built for the purpose of studying continental land surfaces. It has been qualified and calibrated through the implementation of laboratory measurements and in-campaign calibrations, showing a relative polarimetric calibration accuracy better than 0.35 dB, and a cross polarization isolation better than 15 dB, for the full range of elevation angles (from 45° to 90°) relative to the antenna boresight.

Two campaigns were carried out. The first of these involved three qualification flights and was performed in November 2014. It was designed to demonstrate the feasibility of the instrumental concept and to certify GLORI for use as a permanent payload in the French ATR-42 research aircraft. A dedicated campaign was organised in 2015, focusing on airborne observations over agricultural and forest areas, made concurrently with *in situ* measurements of biomass, soil moisture and roughness data. In total, more than 20 h of raw data were collected, including several hours over monitored agricultural and forest areas.

Four types of land surface product can be produced with the GLORI instrument, in both LHCP and RHCP polarization: (1) Delay Doppler maps, providing information related to the Delay-Doppler space distribution of reflected energy; (2) Waveforms measured as the reflected energy at zero Doppler shift with respect to the direct signal; (3) Corrected Interferometric Complex Field, as the ratio of the reflected to direct waveform, corrected for noise and antenna gain and (4) the apparent reflectivity, as the main estimation of the coherently scattered reflected signal. A value between 1 ms and 20 ms can be selected for the coherent integration time, and an incoherent integration time up to several seconds can be used. In the case of the measurements presented in this study, a coherent integration time of 5 ms and an incoherent averaging time of 200 ms were selected, since they provided a good compromise in terms of spatial resolution/sensitivity for all of the studied land surfaces. In the future, more detailed analyses will be needed to determine the optimal coherent integration time (in terms of system performance) for a given type of land surface.

Delay Doppler maps were presented for various types of land use: water, bare soil, corn fields and pine forest. The results demonstrated the instrument’s sensitivity to weak signals, such as those scattered by dense forests. However, its ability to discriminate between coherently and incoherently scattered signals is yet to be demonstrated and improved electromagnetic modelling of these processes is needed, in order to gain a more detailed understanding of the mechanisms involved in the structure of the received signals, especially over areas covered by vegetation.

Various reflectivity maps were produced over the entire study zone, and are shown to have a good dynamic range (at least 20 dB), allowing to distinguish between various types of land surface. The reflected signal intensity was found to vary, as expected, with elevation angle (nearly stable power in LHCP, increasing power with decreasing elevation angle in RHCP).

Measurements were illustrated over two types of fields in agricultural areas: recently planted carrot fields, and mature corn fields. Although the GLORI instrument demonstrated its ability to discriminate between these two types of agricultural vegetation, further studies and modelling are needed in order to validate the results, especially taking into account soil moisture variations.

Measurements were also made over forest stands of various age classes. It was found the instrument can detect changes in forest biomass, with a sensitivity to apparent reflectivity of approximately −30 dB. This sensitivity should allow to estimate forest biomass up to levels of ~200 t/ha.

Overall, the experimental campaigns produced results which validate the GLORI instrument’s potential for land surface characterization, and highlighted the scientific potential of this approach for the analysis of surface scattering properties based on airborne GNSS reflectometry. Future works will include the evaluation of forward models and inversion algorithms, allowing soil moisture or above ground vegetation biomass retrieval performances to be investigated in detail.

## Figures and Tables

**Table 1:** Instrument specifications and capabilities.

Parameter	Instrument Capabilities	Current Set-up
Operating frequency	925 to 2175 MHz (channel by channel selectable)	GPS L1 (1575 ± 2 MHz)
Bandwidth	Up to 40 MHz SSB	4 MHz SSB
Sampling frequency	Up to 65 MSPS	10.0 MSPS (2015), 16.368 MSPS (2014)
Polarization	Zenith: RHCP and LHCP Nadir: LHCP and RHCP	Zenith: RHCP Nadir: LHCP and RHCP
Code/Modulation	All L Band GNSS Open services (following implementation of the coding/modulation scheme)	GPS L1 C/A BPSK
Length of individual files	No minimum, Maximum: storage limited.	15, 30, 36 or 60 s
Calibration scheme	1-to-1 relative channel cross-calibration	1-to-1 relative channel cross-calibration

**Table 2:** Relative calibration stages.

Calibration Stage	Channel 1	Channel 2	Channel 3
Flight mode	ZR	NL	NR
Cal_1	ZR	NR	NL
Cal_2	NL	ZR	NR
Cal_3	NR	NL	ZR

**Table 3:** Description of the regions of interest.

Id	Name	Cover	Latitude	Longitude
Zone 1	Lamasquere	Agricultural area, flat	43.49	1.23
Zone 2	Auch	Agricultural area, hills	43.69	0.74
Zone 3	Nezer	Forest + Agricultural, flat	44.55	−1.02
Zone 4	Marcheprime	Forest + Agricultural, flat	44.71	−0.90
